# Supplementary material for: Revisiting self-interference in Young’s double-slit experiments
Source: Sci Rep. 2023 Jan 18;13:977. doi: 10.1038/s41598-023-28264-1 (PMC9849424; doi:10.1038/s41598-023-28264-1)
Supplement: Supplementary file 1 — Supplementary Information. [file 41598_2023_28264_MOESM1_ESM.pdf]

Supplementary Materials

Title: Revisiting self-interference in Young's double-slit experiments

Authors: Sangbae Kim & Byoung S. Ham*
Affiliation: School of Electrical Engineering and Computer Science, Gwangju Institute of Science and Techology, Gwangju 61005, South Korea
*bham@gist.ac.kr

1. Photon measurements by a single photon detector

Figure S1 shows schematic of photon statistics of the attenuated coherent light (Omicron, PhoxX405-250) for Fig. 2. For this, OD 10 of neutral density filters is used to reach a mean photon number at $\langle n \rangle = 0.04$ (see Methods). All single photon and coincidence measurements are performed by a coincidence counting module (Altera, DE2) via two single photon counting modules (Excelitas SPCM-AQRH 15).

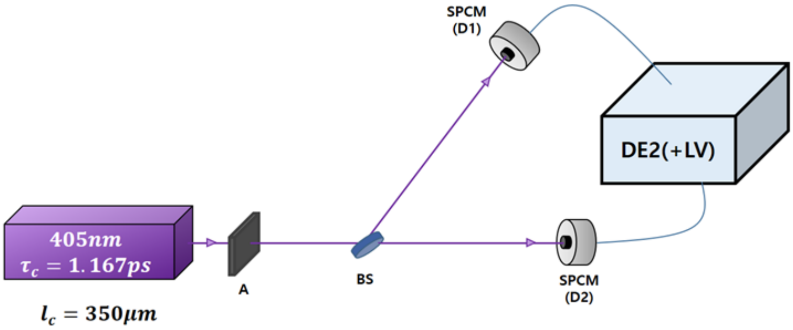

Fig. S1. Schematic of Fig. 2.

Table S1 shows the measurement results of Fig. S1. For this, the data was measured for 600 seconds as shown in Fig. 2. The error rates (standard deviation) are ~1.7% for single photon measurements and 4.5% for coincidence measurements. The higher error in coincidence measurements is due to less data, where the occurrence ratio of doubly bunched photons to single photons is ~1 % as shown in Table S1. The higher order bunched photon rates are negligibly small.

Table S1. Photon statistics for Fig. S1. D1&D2: coincidence detection.

count/s	D1	D2	D1&D2
AVG.	1,024,760	1,039,171	11,752
STDEV.	17,594	17,877	526

2. Photon measurements by a fast digital oscilloscope

Figure S2(a) shows data recorded on the fast digital oscilloscope fed by two single photon detectors in a modified scheme of Fig. S2(b). Due to the memory limitations of the oscilloscope, only 1 ms length data are recorded for two channels of D1 and D2 as shown in Fig. S2(a). For the single-photon number counts in each channel, a Matlab program is used, resulting in 1,011 counts/ms and 1,017 counts/ms for D1 and D2, respectively.

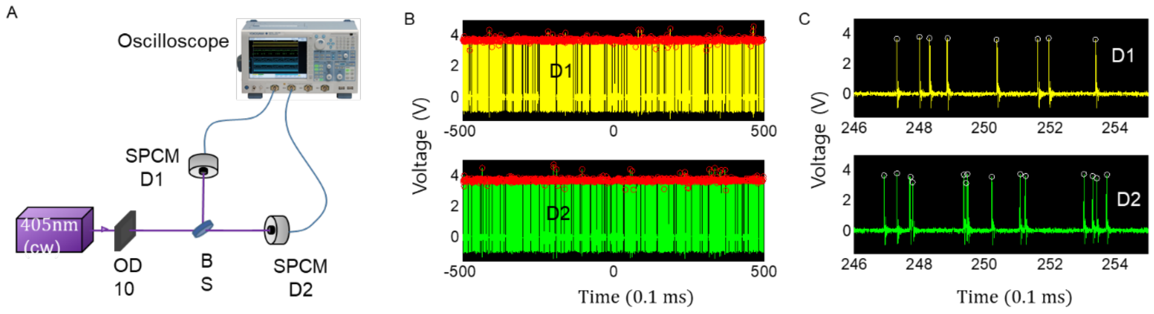

Fig. S2. Single photon counting by a fast digital oscilloscope. (a) A schematic diagram. (b) Recorded single photon streams in both channel of D1 and D2. (c) Expanded data for (b).

Figure 3 shows details of overlapped data counted for coincidence measurements. For 1 ms, we have 11 coincidence data, which is corresponding to ~1% of the single photon rate and similar to the most spontaneous parametric down conversion process-based coincidence measurements.

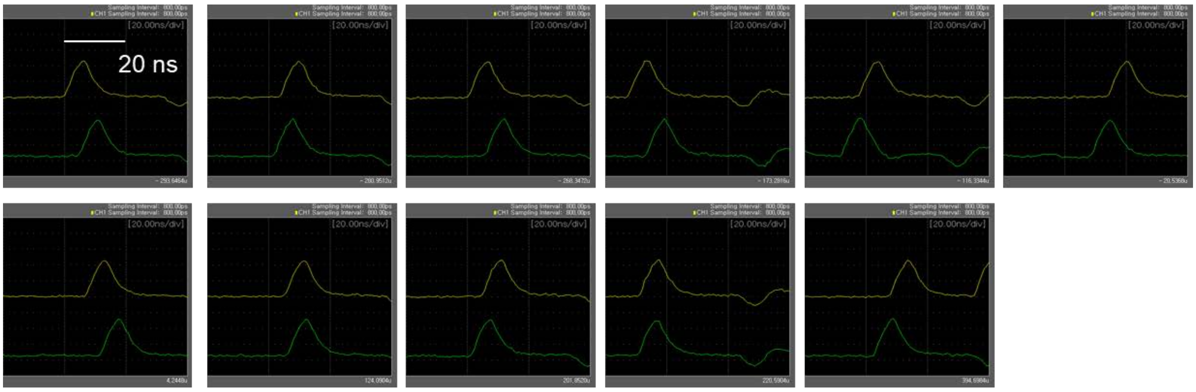

Fig. S3. Coincidence counts in Fig. S2.

For the photon counting in Fig. S2, we programed a Matlab program and confirmed in several local regions as shown in Fig. S3.

3. Visibility calculations from the self-interference data in Fig. 4

Figures S4 and S5 show Gaussian-fit curves applied to the path length-dependent visibilities observed in Fig. 4, respectively. The best-fit lines for those data are nearly coincident with each other as shown in Fig. 5(c) in the main text. The equal relation between visibility and coherence in ref. 29. All data are within the error bars from the best-fit curves, respectively.

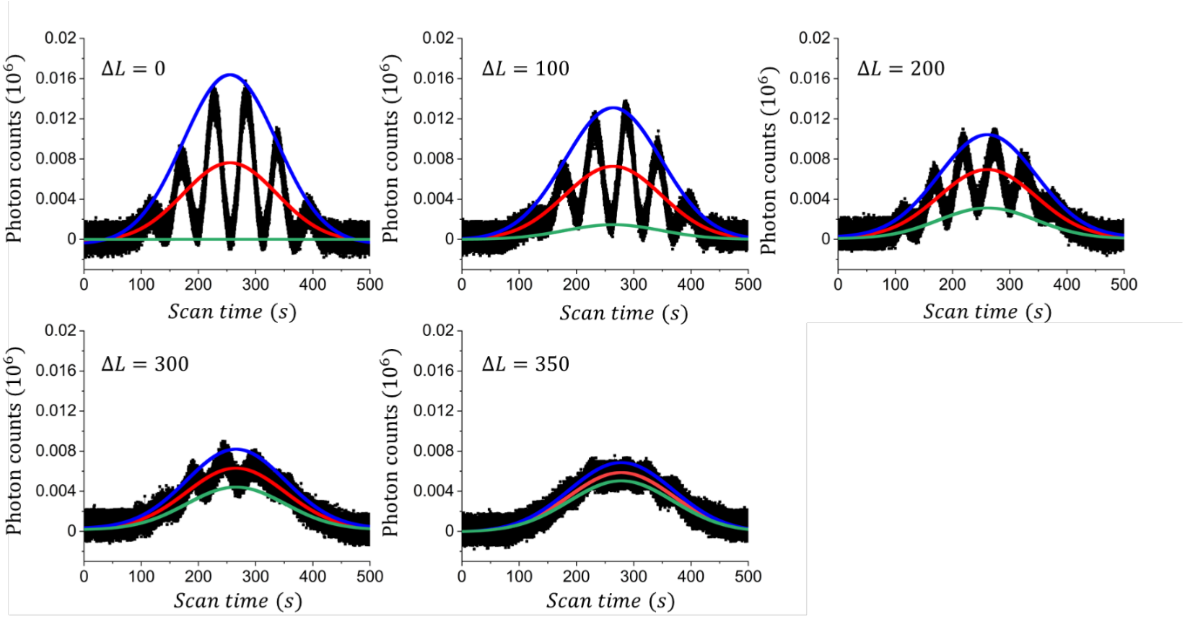

Fig. S4. Gaussian-fit curves for the top panels of Fig. 4.

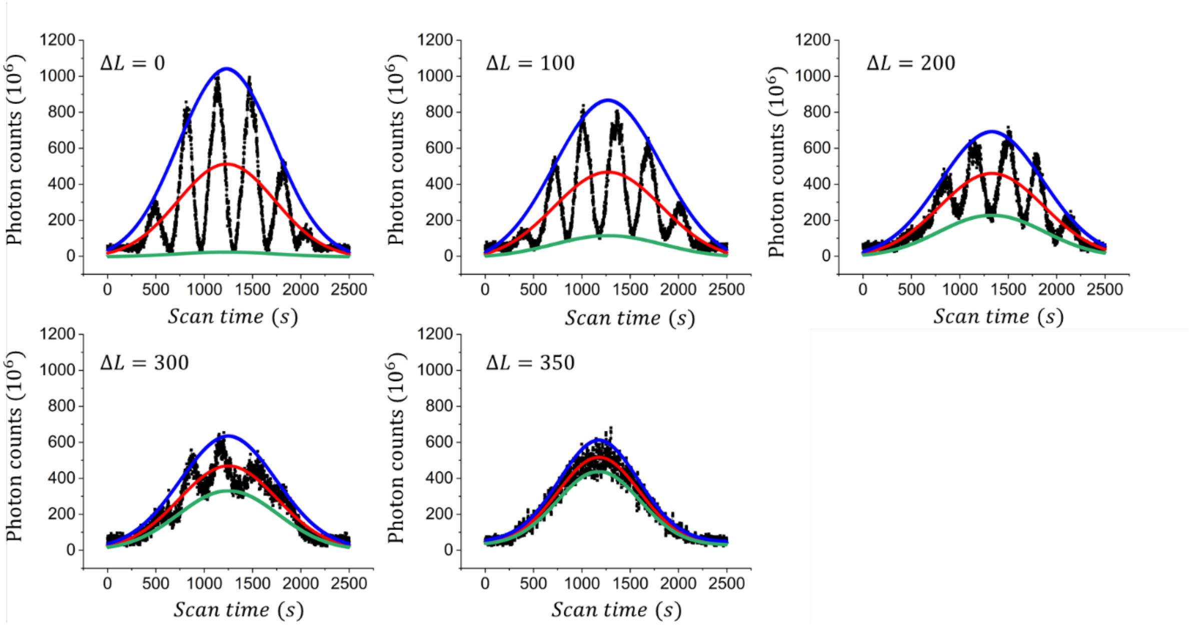

Fig. S5. Gaussian-fit curves for the bottom panels of Fig. 4.

Table S2. Data analysis for Fig. 5(c).

	CW		SP	
Δl	visibility	sigma	visibility	sigma
0	1	0.0223	1	0.02985
100	0.801	0.0155	0.8	0.03147
200	0.539	0.0094	0.525	0.03518
300	0.3	0.0388	0.326	0.06968
350	0.153	0.0397	0.171	0.07351

Source \ ΔL	0	100	200	300	350
CW	1	0.801	0.539	0.3	0.153
SP	1	0.8	0.525	0.326	0.171
CW-fitted	1	0.764	0.528	0.293	0.175
SP-fitted	1	0.768	0.537	0.305	0.190
